# Optimal therapeutic strategies for pineal region lesions

**DOI:** 10.3389/fneur.2023.1261054

**Published:** 2023-12-01

**Authors:** Li-tian Huang, Chun-xi Wang, Tang Li, Sheng-yong Wang, Quan Zhou, Xiaoguang Tong

**Affiliations:** ^1^Clinical College of Neurology, Neurosurgery and Neurorehabilitation, Tianjin Medical University, Tianjin, China; ^2^Department of Neurosurgery, The First Affiliated Hospital of Guangxi Medical University, Guangxi, China

**Keywords:** pineal region lesions, therapeutic strategies, prognostic factors, surgical algorithm, hydrocephalus

## Abstract

**Background:**

The removal of pineal region lesions are challenging, and therapeutic strategies for their removal remain controversial. The current study was conducted to identify the characteristics and the optimal therapeutic strategies for pineal region lesions.

**Methods:**

This retrospective study reviewed the treatments of 101 patients with pineal region lesions, and different characteristics and therapeutic strategies were observed.

**Results:**

There were no statistical differences in the total resection ratio, complications, and prognosis outcomes between the hydrocephalus group and non-hydrocephalus group, except patients in the hydrocephalus group were younger and pediatric patients had an increased level of intracranial infections. Treatments of lesions and hydrocephalus secondary to pineal region lesions were two integral parts to therapeutic strategies. For the management of lesions, germinoma or non-germinoma were diagnosed preoperatively, and resection or diagnostic radiation were chosen to deal with pineal region lesions. Endoscopic-assisted surgery provided a higher total resection rate. For the management of hydrocephalus, endoscopic third ventriculostomy (ETV) had the better therapeutic effect. Additionally, cerebrospinal fluid (CSF) diversion before radiotherapy or resection did not improve prognosis outcome, but it was necessary for patients with severe hydrocephalus. Logistical regression analysis indicated that age, lesion size, reoperation ratio, and intracranial complications were predictors of prognosis outcome.

**Conclusion:**

More attention should be paid to intracranial infections in pediatric patients with hydrocephalus secondary to pineal region lesions, and CSF diversion before radiotherapy or resection did not promote prognosis outcome, but it was necessary for patients with severe hydrocephalus. Age, lesion size, reoperation ratio, and intracranial complications may be the predictors of prognosis outcome. Most importantly, the surgical algorithm for pineal region lesions which was based on preoperatively diagnosis (non-germinoma and germinoma) is useful, especially for developing countries.

## 1 Introduction

The management of pineal region lesions is a challenge, especially in developing countries, due to their location deep in the brain and the vital structures that surround the area. To date, therapeutic strategies for pineal region lesions remain controversial (1). Current strategies for pineal region lesions include surgical resection, radiotherapy, chemotherapy, and biopsy, which are based on clinical features and histologic diagnosis. Recently, surgical resection has become increasingly prevalent for the management of pineal region lesions (2). Indeed, many surgeons believe surgical resection to be a more efficient therapy strategy since compression can be greatly reduced and histologic diagnosis can be achieved (3). However, others consider surgical resection to be a dangerous and, in many cases, unnecessary procedure that has a poor outcome (4). As there is yet no definitive “best practice” procedure to deal with pineal region lesions, we performed a retrospective study to show the characteristics of pineal region lesions, determine suitable predictors for pineal region lesions, and conclude the surgical algorithm for pineal region lesions.

## 2 Materials and methods

### 2.1 Patients and methods

Between 2010 and 2020, 101 patients with pineal region lesions were treated at the First Affiliated Hospital of Guangxi Medical University (Guangxi Province, China). This study included 47 adults and 54 pediatric patients.

Of the 101 patients, 85 were diagnosed with hydrocephalus secondary to pineal region tumors using computed tomography (CT) studies and magnetic resonance imaging (MRI). Tumor markers, cytologic examination of cerebrospinal fluid (CSF), and plasma hormone levels were routinely checked in all enrolled patients. Retrospective data collection was approved by the ethics committee of the First Affiliated Hospital of Guangxi Medical University.

### 2.2 Management

A total of 88 of the 101 patients underwent surgical treatment. Overall, 57 patients were diagnosed as non-germinoma preoperatively using tumor markers, cytologic examination of CSF, plasma hormone levels, and neuroradiologic studies, and 38 of the 57 patients underwent pineal lesion resections. Of those 38 patients, 24 accepted CSF diversion before resection due to severe hydrocephalus and 14 underwent resection directly. Moreover, the other 19 of the 57 patients who were diagnosed as non-germinoma preoperatively declined further resection of the lesions and accepted CSF diversion only. Radiation therapy was conducted on 31 patients who were diagnosed as germinoma preoperatively; of these, 24 patients underwent CSF diversion before radiation therapy due to severe hydrocephalus and 7 patients accepted radiotherapy directly.

### 2.3 Surgical procedure

Seven of the 38 patients underwent microsurgery. As previously reported (5), in the microsurgery group, the Poppen or Krause approach was used after general anesthesia. Next, a bone flap was made and the dura mater opened. Then, the tentorium, adjacent to the straight sinus, was cut and extended to the edge of the tentorium cerebelli, and the tumor was separated from the surrounding tissue. Thirty-one of the 38 patients underwent endoscopic-assisted surgery using the same approaches (Poppen or Krause approach) as the microsurgery group with minor modifications. Briefly, a 0° endoscope was fixed with a machinery-driven arm and introduced into the surgical field to replace the microscope to provide a larger viewing angle of the surgical field, closer observation, and a more panoramic view.

### 2.4 Neuroradiological studies

Lesion size was calculated using preoperatively MR images and evaluated as the tumor with the largest diameter. Postoperative routine imaging examinations were conducted to estimate the effect of treatment. The severity of hydrocephalus was evaluated using neuroradiologic studies and symptomatic and effective hydrocephalus treatment was defined as shrinkage of the brain ventricles (which were enlarged preoperatively) or symptomatic improvement of intracranial hypertension (6). On the first to third postoperative days, CT and MRI tests were performed to evaluate the extent of resection, intracranial hemorrhage, and ischemia. A subtotal resection was defined as any residual tumor revealed in the postoperative MR image (6).

### 2.5 Assessment of prognosis outcome

The modified Rankin Scale (mRS) and Karnofsky Performance Scale (KPS) were used to quantify neurological outcome. A favorable functional outcome was defined as an mRS score ≤ 2 and poor functional outcome as an mRS score ≥ 3 (7).

### 2.6 Statistical analysis

The key features of patients with pineal region lesions were reviewed. Analyses were performed using the Student’s *t*-test, chi-square test, and Fisher’s exact test. SPSS 26.0 (SPSS, Chicago IL, United States) was used for the statistical analysis. Continuous variables are presented as the mean ± standard deviation. Logistic regression analysis of modified Rankin Scale (mRS) scores and receiver operating characteristic curve (ROC) was performed to define the predictors of prognosis outcome. *p* < 0.05 was considered statistically significant.

## 3 Results

### 3.1 General comparison between the hydrocephalus and non-hydrocephalus group

As shown in Table 1, compared with patients in the non-hydrocephalus group, patients in the hydrocephalus group were younger and had a higher pediatric/adult ratio and longer hospital stays. However, there was no statistical significance in postoperative mRS, rate of gross total resection, complications, recurrence of hydrocephalus in the first year, or the reoperation ratio between the two groups.

**Table 1:** General comparison between patients with and without hydrocephalus secondary to pineal region lesions.

	All (*n* = 101)	Hydrocephalus (*n* = 85)	Non-hydrocephalus (*n* = 16)	*p* Value	*N*
Age (years)	21.5 ± 16.3	19.1 ± 15.3	34.4 ± 16.0	**0.0003**	101
Adult/pediatric patients	47/54	34/51	13/3	**<0.0001**	101
Hospital stays (days)	17.9 ± 13.1	19.2 ± 13.4	10.8 ± 8.3	**0.0022**	101
Gross total resection (GTR) / subtotal resection (STR)	29/9	26/8	3/1	0.7467	38
**Major complications**					
Hemorrhage (%)	9 (10.2%)	8 (9.4%)	1 (6.3%)	1	88
Intracranial infection (%)	14 (15.9%)	13 (15.3%)	1 (6.3%)	0.826	88
Persistent hydrocephalus (%)	14 (15.9%)	14 (16.5%)	0	0.271	88
Reoperation number (%)	16 (18.2%)	16 (18.8%)	0	0.210	88
Recurrence of hydrocephalus in first year (%)	18 (22.2%)	18 (25.4%)	0	0.071	81

Values with statistical significance are shown in bold.

### 3.2 General comparison between the pediatric group and adult group

As shown in Table 2, there was no statistical significance between the pediatric group and adult group (*p* > 0.05), except the pediatric group had a higher intracranial infection rate, hydrocephalus ratio, and male-to-female ratio.

**Table 2:** Comparison between adult and pediatric patients with pineal region lesions.

	Adult (*n* = 47)	Pediatric (*n* = 54)	*p* Value
Hydrocephalus (%)	34 (72.3%)	51 (94.4%)	**0.002**
Sex: male/female	32/15	47/7	**0.031**
Gross total resection (GTR) / subtotal resection (STR)	11/3	18/6	1
**Major complications**			
Persistent hydrocephalus (%)	4 (12.9%)	10 (21.7%)	0.324
Hemorrhage (%)	5 (16.1%)	4 (8.7%)	0.526
Intracranial infection (%)	1 (3.2%)	12 (26.1%)	**0.009**
Reoperation number (%)	7 (22.9%)	9 (19.6%)	0.749
Recurrence of hydrocephalus in first year (%)	7 (19.4%)	11 (24.4%)	0.591

Values with statistical significance are shown in bold.

### 3.3 Therapeutic strategy for pineal region lesions

Different therapeutic strategies for pineal region lesions were chosen according to preoperatively classification of lesions (germinoma or non-germinoma). If patients were diagnosed with germinoma, diagnostic radiation would be undertaken, or resection would be considered. Furthermore, if patients had comorbid severe hydrocephalus, CSF diversion would be performed. As shown in Table 3, 88 patients who accepted the treatment were classified into five groups: the CSF diversion before radiotherapy group; radiotherapy directly group; CSF diversion before resection group; resection directly group; and CSF diversion only group. Compared to the patients with radiotherapy or CSF diversion only, patients who underwent lesion resection showed higher postoperative complications, a greater reoperation ratio (31.6%), and poorer prognosis outcomes (a favorable/poor functional outcome of 23/15). Moreover, resection directly significantly reduced the KPS score after surgery (63.6 vs. 69.3). Patients with CSF diversion only had the highest mortality (31.5%) in the first year when compared to patients who underwent radiotherapy or resection. Thus, further treatments of lesions were necessary in patients with CSF diversion. Furthermore, resection was necessary for patients who were diagnosed with non-germinoma preoperatively, though resection had the following shortcomings: higher postoperative complications and a greater reoperation ratio. Additionally, compared to the radiotherapy directly group, CSF diversion before radiotherapy had higher postoperative complications and reoperation ratio, but there was no statistical difference in prognosis outcomes between the two groups (*p* > 0.05). Moreover, there were no statistical differences in total resection rate, postoperative complications, reoperation ratio, and prognosis outcomes between the resection directly group and the CSF diversion before resection group. There were 16 patients who required reoperation due to a deteriorating hydrocephalus or postoperative hemorrhage, and re-CSF diversion, hematoma aspiration, and residual tumor resection were the main methods of reoperation.

**Table 3:** Therapeutic strategies for pineal region lesions.

Preoperative diagnosis of germinoma?	Therapeutic strategies	Number	Adult/pediatric patients	Male/female	Post/pre operative KPS	Postoperative complications	Reoperation number	GTR/STR	Favorable/poor functional outcome	Recurrence of hydrocephalus (lesions)	Mortality in first year
Yes	CSF diversion before radiotherapy	24	12/12	19/5	82.2/75.6	1 Hemorrhage, 1 Persistent Hydrocephalus, and 4 Intracranial infections	1 VPS	–	20/4	4 (5)	2
	Radiotherapy directly	7	5/2	5/2	87.5/87.1	None	0	–	5/0	0 (0)	0
No	CSF diversion before resection	24	7/17	21/3	67.1/70	3 Hemorrhage, 7 Persistent Hydrocephalus, and 6 Intracranial infections	3 VPS, 2 EVD, and 1 ETV	18/6	14/10	8 (6)	3
	Resection directly	14	7/7	10/4	63.6/69.3	2 Hemorrhage, 6 Persistent Hydrocephalus, and 3 Intracranial infections	4 VPS, 1 EVD + Hematoma aspiration, and 1 EVD + Residual tumor resection	11/3	9/5	5 (4)	2
	CSF diversion only	19	16/3	8/11	71.6/65.8	3 Hemorrhage, 5 Persistent Hydrocephalus, and 1 Intracranial infection	3 EVD	–	13/6	1 (5)	6

CSF, cerebrospinal fluid; KPS, Karnofsky Performance Scale; GTR, gross total resection; STR, subtotal resection; VPS, ventriculoperitoneal shunt; ETV, endoscopic third ventriculostomy; EVD, external ventricular drainage.

**Figure 1:**
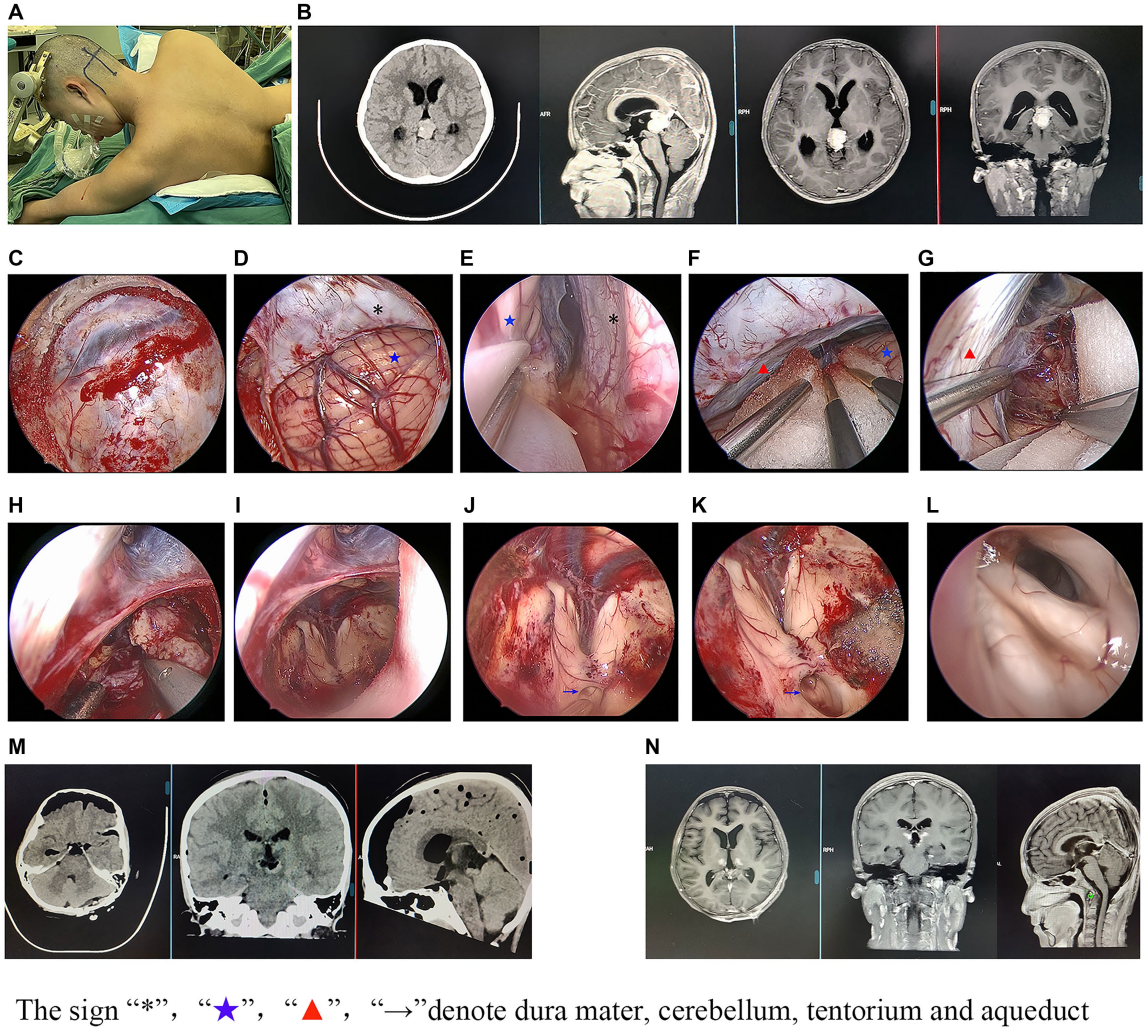
Illustrative case of endoscopic-assisted surgery.

For patients who were diagnosed with non-germinoma preoperatively, lesion resections were undertaken. The clinical surgical modalities and prognosis of the 38 patients who underwent lesion resection are shown in Table 4. We found that gross total resection was achieved in 29 patients (76.3%) while subtotal resection was found in 9 patients (23.7%). Compared with the subtotal resection cases, the total resection cases had a lower reoperation ratio (20.6% vs. 66.7%, *p* = 0.016) and better prognosis outcome (better postoperative mRS scores, less recurrence of hydrocephalus/lesions, and lower mortality in the first year). Moreover, compared with the microsurgery cases, the endoscopic-assisted surgery cases (Figure 1) had a significantly higher total resection ratio (81.7% vs. 28.6%, *p* = 0.004) and less recurrence of hydrocephalus in the first year (32% vs. 71.4%). However, endoscopic-assisted surgery could not significantly reduce the recurrence rate (20.7% vs. 42.9%, *p* = 0.33) of lesions in the first year; a total nine of 38 patients suffered from recurrence of lesion in the first year and further statistical analysis showed that larger lesions size (*p* = 0.039) and a lower gross total resection ratio (*p* = 0.05) may be the possible causes for lesion recurrence. Three patients (cases 10, 22, and 38 in Table 4) were diagnosed with non-germinoma preoperatively and accepted lesion removal but the final pathology was germinoma and thus the misdiagnosis rate was 8%.

**Table 4:** Clinical, surgical modalities, and prognosis of patients who were diagnosed as non-germinoma preoperatively.

Case no.	Sex/age (yrs)	Hydrocephalus (degree of hydrocephalus)	Maximum diameter size of lesions (mm)	Surgical modality	ES/ MS	Outcome	Main complication	Reoperation	Preop mRs score	Postop mRs score	Pathology	Recurrence of hydrocephalus?
**CSF diversion before resection (adult)**												
1	M/20	Yes (Severe)	27	ETV + Resection	ES	Total	No	No	2	1	Mature teratoma	No
2	M/18	Yes (Severe)	28	ETV + Resection	ES	Total	No	No	2	1	Pineal parenchymal tumor	No
3	M/21	Yes (Severe)	39	VPS + Resection	MS	Subtotal	Hemorrhage	EVD	5	5	Choriocarcinoma	Yes
4	M/18	Yes (Severe)	29	ETV + Resection	ES	Total	No	No	1	1	Mature teratoma	Yes
5	M/28	Yes (Severe)	16	EVD + Resection	ES	Total	Mild-hydrocephalus	No	4	1	Pineal parenchymal tumor	Yes
6	M/33	Yes (Severe)	30	VPS + Resection	ES	Total	Mild-hydrocephalus	No	2	2	Germinoma	No
7	F/33	Yes (Severe)	41	EVD + Resection	ES	Total	No	No	1	1	Meningioma	No
**CSF diversion before resection (pediatric patients)**												
8	M/17	Yes	42	ETV + Resection	ES	Total	No	No	3	1	Low-grade glioma	No
9	F/1	Yes	40	ETV + Resection	MS	Total	Mild-hydrocephalus	No	2	3	Cyst	Yes
10	M/6	Yes	17	ETV + Resection	ES	Total	No	No	1	1	Germinoma	No
11	M/14	Yes	32	ETV + Resection	ES	Total	Hemorrhage, hydrocephalus	EVD	3	3	Pineal parenchymal tumor	Yes
12	F/15	Yes	71	EVD + Resection	MS	Subtotal	Intracranial infection	No	2	2	Low-grade glioma	Yes
13	M/7	Yes	58	ETV + Resection	ES	Subtotal	No	No	3	4	Pineal parenchymal tumor	No
14	M/8	Yes	52	EVD + Resection	MS	Total	No	No	5	1	Teratoma	No
15	M/6	Yes	27	VPS + Resection	MS	Subtotal	Hemorrhage, hydrocephalus, intracranial infection	VPS	1	4	Pineal parenchymal tumor	Yes
16	M/0.5	Yes	31	ETV + Resection	ES	Total	Hydrocephalus	VPS	2	2	Medulloblastoma	No
17	M/15	Yes	34	ETV + Resection	ES	Subtotal	Hydrocephalus, intracranial infection	VPS	3	5	Mixed germ cell tumors	Yes
18	M/5	Yes	27	EVD + Resection	MS	Subtotal	Intracranial infection	No	4	2	Teratoma	No
19	M/14	Yes	34	ETV + Resection	ES	Total	Hydrocephalus, intracranial infection	No	2	4	Pineoblastoma	No
20	M/9	Yes	28	ETV + Resection	ES	Total	No	No	3	2	Pineal parenchymal tumor	No
21	M/12	Yes	36	ETV + Resection	ES	Total	Intracranial infection	No	2	3	Seminoma	No
22	M/17	Yes	34	ETV + Resection	ES	Total	Intracranial infection	No	2	3	Germinoma	No
23	M/8	Yes	40	VPS + Resection	ES	Total	Intracranial infection	No	2	5	Germinoma and Teratoma	No
24	M/16	Yes	28	VPS + Resection	ES	Total	No	No	3	2	Pineal parenchymal tumor	No
**Resection directly (adult)**												
25	F/66	Yes (Severe)	26	Resection directly	ES	Total	Hydrocephalus	VPS	4	4	Pineal parenchymal tumor	Yes
26	M/31	Yes (Severe)	66	Resection directly	ES	Total	Hemorrhage, Hydrocephalus	EVD + Hematoma aspiration	3	6	Pineal parenchymal tumor	Yes
27	M/22	Yes (Mild–moderate)	24	Resection directly	ES	Subtotal	Hydrocephalus	ETV	2	3	Low-grade glioma	No
28	F/52	No	28	Resection directly	ES	Total	No	No	1	1	Meningioma	No
29	M/22	No	28	Resection directly	ES	Total	No	No	2	2	Pineal parenchymal tumor	No
30	F/30	Yes (Mild–moderate)	33	Resection directly	ES	Total	Hydrocephalus, intracranial infection	EVD	3	2	Pineal parenchymal tumor	No
31	F/52	No	55	Resection directly	ES	Subtotal	No	No	5	5	Pineal parenchymal tumor	No
**Resection directly (pediatric patients)**												
32	M/11	Yes	60	Resection directly	ES	Total	Hydrocephalus	VPS	3	4	Teratoma and Yolk Sac Tumor	Yes
33	M/8	Yes	50	Resection directly	MS	Subtotal	Hydrocephalus	VPS	5	2	Teratoma	Yes
34	M/15	Yes	23	Resection directly	ES	Total	No	No	1	1	Mixed germ cell tumors	No
35	M/9	Yes	20	Resection directly	ES	Total	No	No	2	1	Teratoma	No
36	M/9	Yes	25	Resection directly	ES	Total	Intracranial infection	No	3	2	Teratoma	No
37	M/1	Yes	27	Resection directly	ES	Total	Hemorrhage, hydrocephalus	VPS	3	3	Pineal parenchymal tumor	Yes
38	M/10	Yes	17	Resection directly	ES	Total	No	No	2	2	Germinoma	No

VPS, ventriculoperitoneal shunt; ETV, endoscopic third ventriculostomy; EVD, external ventricular drainage, mRS, modified Rankin Scale.

### 3.4 Therapeutic strategies for hydrocephalus secondary to pineal regions lesions

If patients had comorbid severe hydrocephalus, treatment for hydrocephalus would be undertaken. In order to identify the optimal therapeutic strategies for hydrocephalus secondary to pineal regions lesions, the comparison of the four strategies [ventriculoperitoneal shunts (VPS), endoscopic third ventriculostomy (ETV), external ventricular drainage (EVD), and direct removal of lesions] for hydrocephalus was also made to further clarify the optimal therapeutic strategies. As shown in Table 5, the direct removal of the lesions group and EVD group had the lowest remission rate of hydrocephalus (54.5% and 77.8%) and highest reoperation ratio (54.5% and 55.6%) among the four groups. There were no statistical differences in the remission rate of hydrocephalus and the reoperation ratio between the VPS and ETV groups (*p* > 0.05). However, the VPS group had the highest rate of postoperative hemorrhage (22.2%). The EVD group had the worst preoperatively condition (lowest preoperatively KPS score), but the KPS scores were improved significantly postoperatively. However, in the EVD group, seven patients needed convert to a permanent CSF diversion because of deteriorating hydrocephalus after removal of the drain. Thus, ETV maybe the better therapeutic strategy for hydrocephalus secondary to pineal regions lesions.

**Table 5:** Four therapeutic strategies for pineal region lesions in our center.

Therapeutic strategies for pineal region lesions	Number	Adult/pediatric patients	Male/female	Post/pre operative KPS	Postoperative complications	Reoperation number	Relief/no relief	Favorable /poor functional outcome	Recurrence of hydrocephalus	Mortality in first year
Ventriculoperitoneal shunt (VPS)	27	16/11	20/7	72.6/71.5	6 Hemorrhage, 7 Persistent Hydrocephalus, and 4 Intracranial infections	1 VPS, 3 EVD	20/7	20/7	7	5
Endoscopic third ventriculostomy (ETV)	27	8/19	24/3	73.70/72.96	1 Hemorrhage, 6 Persistent Hydrocephalus, and 6 Intracranial infections	2 VPS, 2 EVD	21/6	17/10	4	0
Direct removal of lesions (DRL)	11	4/7	8/3	64.55/76.36	1 Hemorrhage, 6 Persistent Hydrocephalus, and 2 Intracranial infections	4 VPS, 1 EVD + Hematoma aspiration, and 1 EVD + Residual tumor resection	6/5	7/4	5	3
External ventricular drainage (EVD)	9	2/7	9/0	78.89/46.67.3	None	4 VPS, 1 EVD, and 2 gave up further treatment	2/7	7/2	-	-

CSF, cerebrospinal fluid; KPS, Karnofsky Performance Scale; GTR, gross total resection; STR, subtotal resection; VPS, ventriculoperitoneal shunt; ETV, endoscopic third ventriculostomy; EVD, external ventricular drainage.

### 3.5 The possible predictors of prognosis outcomes

**Figure 2:**
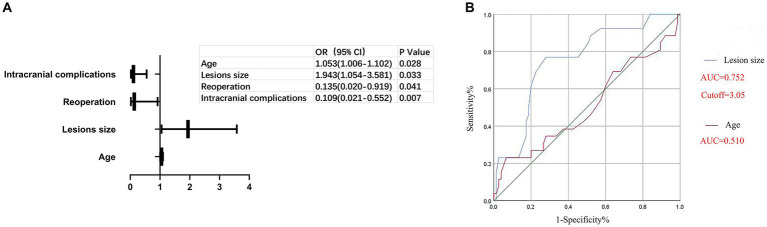
Predictors for the prognosis outcome.

In order to evaluate the prognosis outcomes, patients who underwent surgical treatment were assigned to a favorable functional outcome group and poor functional outcome group according to their mRS scores. Compared with the poor functional outcome group, the favorable functional outcome group had better pre/postoperative KPS scores, shorter hospital stays, lower lesion resections rate, less surgical complications, lower reoperation ratios, and less recurrence of hydrocephalus and mortality in the first year (Supplementary Table S1). Further logistic regression analysis of the mRS scores was performed to define the predictors of prognosis outcome. The analysis indicated that age, lesion size, reoperation ratio, and intracranial complications were the predictors of prognosis outcome, as shown in Figure 2A. Additionally, the receiver operating characteristic curve (ROC) further indicated that a lesion size bigger than 3.05 cm was an independent predictor of poor prognosis, as shown in Figure 2B. Lesion size had high diagnosis value for predictors of prognosis and the Area Under Curve (AUC) of lesion size was 0.752 while age showed low diagnosis value for predictors and the AUC of age was 0.510.

## 4 Discussion

### 4.1 The influencing factors of total resection

With the development of micro-anatomy and micro-neurosurgery, decreasing mortality rates and complications have been found following surgical resection (8). The purpose of surgical resection includes acquiring the histologic diagnosis and removing the lesions (9). Similar to previous reports (10), in our study, we found that total resection was correlated with a better prognosis outcome; furthermore, we clarified the relationship between the total resection ratio and endoscopic-assisted surgery and the relationship between the total resection ratio and CSF diversion before resection as shown in the following:

1. Endoscopic-assisted surgery had the higher total resection ratio. With the aid of endoscopy, a bright, wide-angled panoramic surgical field and close observation view were provided. Thus, the exposure and dissections of the lesions was greatly facilitated (11, 12). Moreover, endoscopic-assisted surgery has been shown to reduce postoperative hemorrhages due to the total resection of the lesion, which improves the recovery rate of the hydrocephalus by reopening the aqueduct (13). In our study, the total resection ratio of endoscopic-assisted surgery and microsurgery were 81.7% and 28.6%, respectively.
2. CSF diversion before resection had little effect on the total resection rate. Many researchers believe that retention of the hydrocephalus before lesion removal may lead to a higher radical resection rate because the shape of the ventricles is maintained and collapse of the ventricles avoided, which leads to better tumor exposure and improved radical tumor resection (14, 15). However, emergent CSF diversion is necessary to manage deteriorating hydrocephalus. In our study, we found that CSF diversion before resection had little effect on the total resection rate (Tables 3, 4), particularly in endoscopic-assisted surgery. We believe that there are several possible explanations as to why this occurred. First, during endoscopic-assisted surgery, the most popularly used position is the lateral prone position and, as a result, a sufficient corridor is maintained due to the natural sagging of the cerebellum, which may provide increased exposure of the lesion (16). Furthermore, many surgeons are of the opinion that the cerebellum naturally sags and the complete relaxation of the brain is a key factor for successful surgery (12, 17). Indeed, hydrocephalus may reduce the relaxation of the brain and may lead to herniation of the tentorial notch (11, 18). In addition, the larger viewing angle of the endoscope may also reduce the blind zone of the surgical field and improve the total resection rate of lesions (19). Thus, CSF diversion before resection did not reduce the total resection rate.

### 4.2 The influencing factors of prognosis outcomes

Controversy remains regarding the possible predictors of prognosis outcome. Many researchers believe that age and lesion location are the main predictors of prognosis outcome (20, 21), whereas others have shown that lesions size and histology are closely related to prognosis outcome (22). Moreover, the extent of surgical resection has been regarded as vital for prognosis outcome (13, 19). In the current study, we found that age, lesions size, the reoperation ratio, and intracranial complications were the predictors of prognosis outcome (Figure 1). As in previous reports (19), a larger lesion size has been found to increase brain compression, resulting in decreased lesion exposure and resection, which may lead to a poor prognosis outcome, as shown in Figure 1. Deteriorating hydrocephalus and postoperative hemorrhage were the main reasons for reoperation, both of which were also the main intracranial complications postoperatively (Table 3). Higher rates of deteriorating hydrocephalus and postoperative hemorrhage could most likely cause worse prognosis outcomes.

### 4.3 The prevention of intratumoral hemorrhage

Intratumoral hemorrhage could lead to reoperation and usually results in disaster. Intratumoral hemorrhage of pineal region lesions was uncommon and was characterized by gaze paresis, ataxia, and intracranial hypertension (23, 24). Unfortunately, the mortality rate of intratumoral hemorrhage of pineal region lesions was high (25). In our study, we found five patients who had intratumoral hemorrhages: one patient suffered from intratumoral hemorrhage when he underwent radiotherapy, three patients suffered from intratumoral hemorrhage after VPS, and one case suffered from intratumoral hemorrhage after tumor recurrence. Lesion resection was performed in two patients, EDV was performed in one patient, and conservative management was taken in two patients to deal with intratumoral hemorrhage. Only one patient survived for more than 1 year. Some researchers believe that irradiation, tumor growth characteristics, hypertension, craniocerebral trauma, anticoagulant therapy, and idiopathic causes are the causes of intratumoral hemorrhage (23, 26). Moreover, as previously reported, VPS can lead to a sudden decrease in intracranial hypertension (ICP) which may be the cause of intratumoral hemorrhages. Disturbance of the dynamic balance caused by ventricular tapping could lead to the increase in cerebral blood flow and vascular congestion and which could trigger intratumoral hemorrhages, particularly in highly vascularized tumors (26, 27). We agree with this view. We believe that the keys to preventing intratumoral hemorrhages are maintaining appropriate intracranial pressure after CSF diversion and preventing a sudden decrease in ICP, especially in highly vascularized tumors such as seminoma and malignant germ cell tumors. We preferred used ETV rather than VPS to treat hydrocephalus, because ETV could reduce ICP smoothly when compared with VPS. Additionally, three patients suffered from intratumoral hemorrhage after VPS in our study which seemed to further prove this conclusion.

Moreover, we believed that the surgical treatment of apoplectic pineal lesions and hydrocephalus secondly to hemorrhage were two key factors in the management of intratumoral hemorrhage, and the conservative management of intratumoral hemorrhage may lead to worse prognosis, because ICP and compression of the brain were the predominant causes of death.

### 4.4 The optimal therapeutic strategy for hydrocephalus secondary to pineal region lesions

Deteriorating hydrocephalus was very common in patients with pineal region lesions. VPS, ETV, EVD, or the direct removal of lesion was always performed to deal with hydrocephalus (1, 13). VPS was widely used at an early stage. However, the lifelong catheter, shunt-related complications, and increased cancer cell dissemination made it limited and the shunt related complications also could be found in our study (Table 5) (13). EVD was a temporary way to manage emergent hydrocephalus though it was simple and effective solution. However, the high infection rate and reoperation ratio of hydrocephalus made it restricted (28). In our study, seven of nine patients needed convert to a permanent CSF because of deteriorating hydrocephalus after removal of the drain (Table 5). We always took EVD in surgery to make the complete relaxation of the brain tissues. Additionally, EVD was a rapid, effective and temporary treatment of hydrocephalus, and we always took EVD to treat fatal hydrocephalus in our center, because EVD could be made quickly. Some people believe that the direct removal of lesions is a good choice for the treatment of hydrocephalus because, in most cases, direct removal of the lesions can relieve the hydrocephalus and further treatment of hydrocephalus is not required after lesion resection (2, 6). However, we do not altogether agree with this opinion. Firstly, about 12%–81% of patients needed extra CSF diversion after the direct removal of the lesions and about 2.1% of adults and 10%–40% of pediatric patients had new onset hydrocephalus postoperatively (19, 29). Moreover, the direct removal of lesions could lead to a lower remission rate of hydrocephalus and higher reoperation as shown in our study (Table 5). Thus, whether we should adopt the direct removal of lesions or choose CSF diversion to treat hydrocephalus secondary to pineal region lesions is worth continuing paying attention to. In our opinion, ETV was the better therapeutic strategy for hydrocephalus secondary to pineal regions lesions because ETV was equally effective as VPS, and ETV could avoid the shortcomings of VPS and the direct removal of the lesions (13, 30), though ETV had the shortcoming of a higher rate of failure in earlier (31). Thus, ETV has been performed as an alternative to VPS in our center in recent years.

### 4.5 Surgical algorithm for pineal region lesions

**Figure 3:**
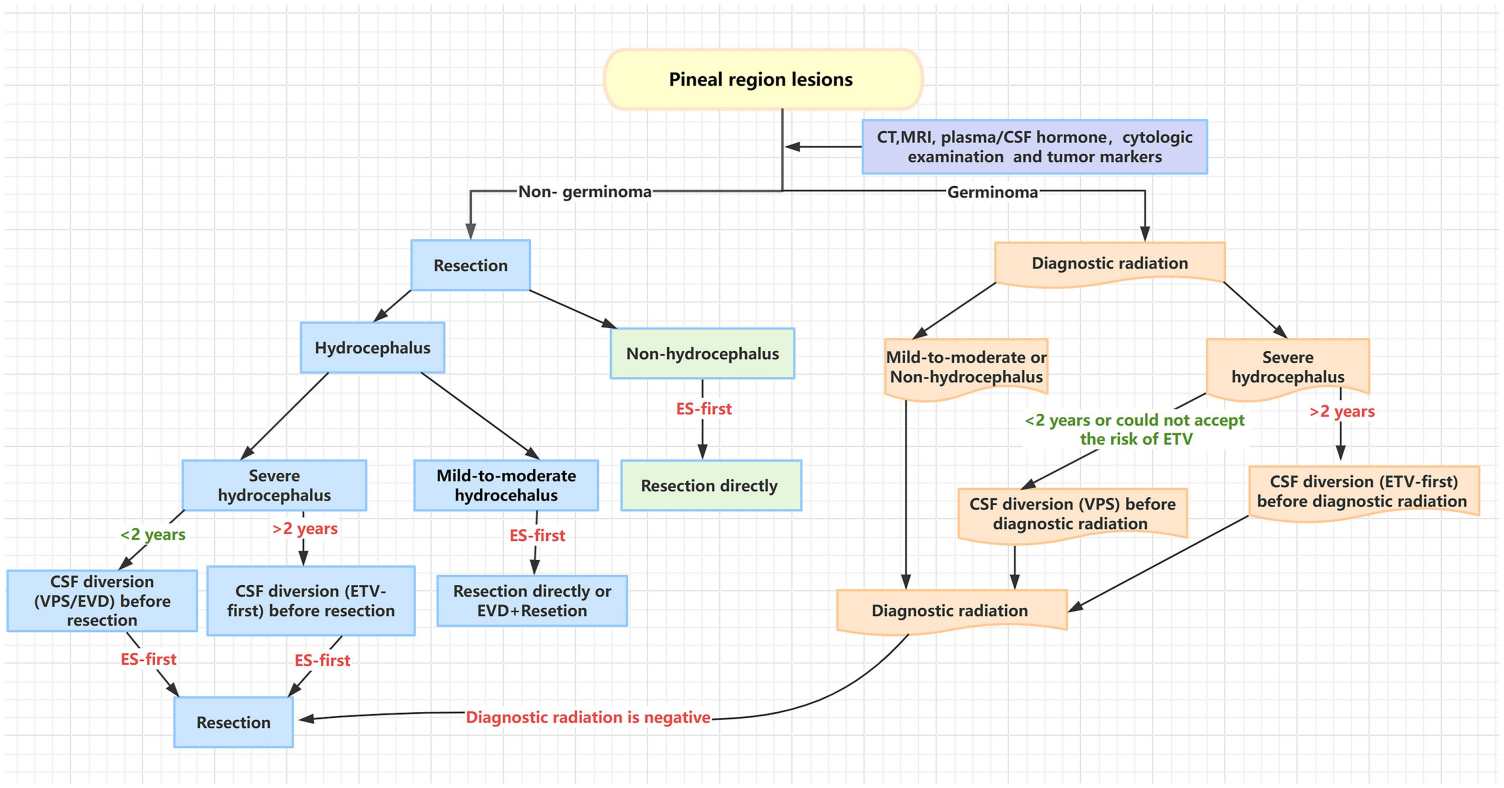
Algorithm of strategies for treating pineal region lesions.

As shown in Figure 3, we sum the experiences and relevant reports and provide the surgical algorithm for pineal region lesions. Based on tumor markers, cytologic examination of CSF, plasma hormone levels, and neuroradiologic studies preoperatively, pineal region lesions were classified into two main categories preoperatively: non-germinoma and germinoma. Diagnostic radiation could be taken to treat germinoma while lesion removal would be chosen to deal with non-germinoma. If patients had comorbid severe hydrocephalus, we preferred to undertake CSF diversion (ETV) before resection or radiotherapy to relieve intracranial hypertension or make resection technically easier by reducing brain swelling. However, it is worth noting that CSF diversion before resection is not the preventative treatment of hydrocephalus after surgery because there is no clear evidence to prove that CSF diversion before resection can reduce hydrocephalus after surgery (32, 33). Notably, the ETV success rate for young infants (< 2 years of age) was low and permanent CSF diversion (VPS) before resection/radiotherapy or temporary CSF diversion (EVD) were better choices for young infants with severe hydrocephalus (34), though some researchers held different opinions (35). Additionally, VPS before resection was also performed in patients who could not accept the risk of ETV. Moreover, for patients undergoing diagnostic radiation, if pineal region lesions cannot be reduced by diagnostic radiation, further resection would be recommended. For patients having lesion resection, endoscopic-assisted surgery could be recommended as a priority due to endoscopic-assisted surgery having a significantly higher total resection ratio and less recurrence hydrocephalus. There is no doubt that a lack of biopsies preoperatively will be controversial for this surgical algorithm because biopsies can provide a histologic diagnosis preoperatively and one-third of such patients will not require an operative resection (36). However, the risks of biopsy, extensive tissue heterogeneity, and mixed cell populations of pineal region lesions make the use of biopsies limited (37). Additionally, the lack of suitable equipment and technology in developing countries further limits the use of biopsies. Furthermore, biopsies sometimes cannot provide sufficient tissue for an accurate histologic diagnosis, which may lead to a misdiagnosis (38). Some researchers believe that tumor markers, cytologic examination of CSF, plasma hormone levels, and CT and MRI images could replace a biopsy and provide accurate preoperative diagnosis (39, 40). As in previous reports, if blood and/or CSF markers are positive (germinoma), diagnostic radiation is performed. Conversely, the surgical excision of pineal region lesions remains the standard when blood and/or CSF markers are negative (non-germinoma) (39), and we quite agree with these treatment strategies. In our study, we find the preoperatively diagnostic method which is based on tumor markers, cytologic examination of CSF, plasma hormone levels, and neuroradiologic studies shows an accurate diagnosis rate. As shown in Table 4, based on this diagnosis method, the misdiagnosis rate in our center was just 8%. Thus, the surgical algorithm for pineal region lesions in our study is useful though it has some drawbacks. It is particularly suitable for treatment centers in developing countries due to its good and economical operability.

## 5 Limitations

Our study has some limitations. Firstly, this study was a retrospective review. Secondly, all data were collected from a single center. Lastly, the surgeries were performed by different neurosurgeons.

## 6 Conclusion

More attention should be paid to intracranial infections in pediatric patients with hydrocephalus secondary to pineal region lesions, and CSF diversion before radiotherapy or resection did not promote prognosis outcome but it was necessary for patients with severe hydrocephalus. Age, lesion size, reoperation ratio, and intracranial complications may be the predictors of prognosis outcome. Most importantly, the surgical algorithm for pineal region lesions which was based on preoperatively diagnosis (non-germinoma and germinoma) is useful, especially in developing countries.

## Data availability statement

The raw data supporting the conclusions of this article will be made available by the authors, without undue reservation.

## Ethics statement

The studies involving humans were approved by the ethics committee of the First Affiliated Hospital of Guangxi Medical University. The studies were conducted in accordance with the local legislation and institutional requirements. Written informed consent for participation was not required from the participants or the participants’ legal guardians/next of kin in accordance with the national legislation and institutional requirements.

## Author contributions

L-tH, QZ, and XT contributed to the conception and design of the study. All authors contributed to the acquisition and analysis of data and helped to make draft the text and create the figures.
