# Supplementary material for: Optimal therapeutic strategies for pineal region lesions
Source: Front Neurol. 2023 Dec 1;14:1261054. doi: 10.3389/fneur.2023.1261054 (PMC10722988; doi:10.3389/fneur.2023.1261054)
Supplement: Supplementary file 1 [file Data_Sheet_1.docx]

Supplementary Table 1. General comparison between favorable functional outcome group and poor functional outcome group

|  | All  (n =88) | Favorable functional outcome (mRS≤2)  (n =63) | Poor functional outcome (mRS≥3)  (n = 25) | P Value | N |
| --- | --- | --- | --- | --- | --- |
| Age (years) | 20.33 ± 15.05 | 19.58 ± 13.15 | 22.22 ±19.23 | 0.4612 | 88 |
| Sex: Male/Female | 71/17 | 50/13 | 21/4 | 0.619 | 88 |
| Adult/ Pediatric | 39/49 | 29/34 | 10/15 | 0.607 | 88 |
| Hospital stays(days) | 19.55 ± 13.12 | 17.49 ± 11.49 | 24.72 ± 15.62 | **0.0189** | 88 |
| Preoperative KPS | 71.36± 19.37 | 74.92±16.84 | 62.40 ±22.60 | **0.0056** | 88 |
| Lesions size（cm） | 3.114±1.254 | 2.784±1.083 | 3.944±1.292 | **<0.0001** | 88 |
| Postoperative KPS | 73.07 ±25.43 | 86.98 ±5.575 | 38.00±21.79 | **<0.0001** | 88 |
| Postoperative mRS | 2.193± 1.380 | 1.429±0.5598 | 4.120±0.8327 | **-** | 88 |
| lesion resections | 38 | 23 | 15 | **0.045** | 88 |
| Gross total resection (GTR))/ Subtotal resection (STR) | 29/9 | 19/4 | 10/5 | 0.436 | 38 |
| Endoscopic-assisted surgery (ES)/ Microsurgery (MS) | 31/7 | 19/4 | 12/3 | 1 | 38 |
| Major complications | - | - | - | - |  |
| Postoperative hemorrhage（%） | 9 (10.2%) | 1 (1.6%) | 8(32%) | **<0.0001** | 88 |
| Intracranial infection（%） | 14 (15.9%) | 8 (12.7%) | 6 (24%) | 0.191 | 88 |
| Pneumonia（%） | 19 (21.6%) | 11 (17.5 %) | 8 (32%) | 0.135 | 88 |
| Hydrocephalus | 77 | 54 | 23 | 0.421 | 77 |
| Remission number of hydrocephalus (%) | 63 (84.1%) | 51(94.4%) | 12 (48%) | **<0.0001** | 77 |
| Reoperation number (%) | 16(18.2%) | 5(7.9%) | 11(44%) | **<0.0001** | 88 |
| Recurrence or progressive of hydrocephalus in first year (%) | 18(22.2%) | 10(16.1%) | 8(42.1%) | **0.017** | 81 |
| Recurrence or progressive of lesions in first year (%) | 19(23.5%) | 15(24.2%) | 4(21.1%) | 0.955 | 81 |
| Mortality in first year（%） | 12(14.8%) | 2 (3.2%) | 10 (52.6 %) | **<0.0001** | 81 |

For numeric variables, values are mean± standard deviation. Values with statistical significance are shown in bold. KPS, Karnofsky Performance Scale; mRS, modified Rankin Scale.
